# Rapid Urban Mapping Using SAR/Optical Imagery Synergy

**DOI:** 10.3390/s8117125

**Published:** 2008-11-12

**Authors:** Christina Corbane, Jean-François Faure, Nicolas Baghdadi, Nicolas Villeneuve, Michel Petit

**Affiliations:** 1 ESPACE Unit, Institut de Recherche pour le Développement, Maison de la télédétection, 500 rue JF Breton, F34093 Montpellier cedex 5, France; E-mail: michel.petit@ird.fr; 2 ESPACE Unit, Institut de Recherche pour le Développement, Centre IRD de Cayenne, Route de Montabo, PO Box 165, F97323, Cayenne, Guyane Française; E-mail: jean-francois.faure@cayenne.ird.fr; 3 CEMAGREF, UMR TETIS, 500 rue François Breton, 34093 Montpellier cedex 5, France; E-mail: baghdadi@teledetection.fr; 4 ESPACE Unit, Institut de Recherche pour le Développement, Campus universitaire du Moufia, PO Box 172, 97492, Sainte-Clotilde, île de la Réunion; E-mail: nicolas.villeneuve@ird.fr

**Keywords:** SAR sensors, optical sensors, texture analysis, fuzzy K-means classification, information fusion, rapid urban mapping

## Abstract

This paper highlights the potential of combining Synthetic Aperture Radar (SAR) and optical data for operational rapid urban mapping. An algorithm consisting of a completely unsupervised procedure for processing pairs of co-registered SAR/optical images is proposed. In a first stage, a texture analysis is conducted independently on the two images using eight different chain-based Gaussian models. In a second stage, the resulting texture images are partitioned by an unsupervised fuzzy K-means approach. Finally, a fuzzy decision rule is used to aggregate the results provided by the classification of texture images obtained from the pair of SAR and optical images. The method was tested and validated on images of Bucharest (Romania) and Cayenne (French Guiana). These two study areas are of different terrain relief, urban settlement structure and land cover complexity. The data set included Radarsat-1/ENVISAT and SPOT-4/5 images. The developed SAR/optical information fusion scheme improved the capabilities of urban areas extraction when compared with the separate use of SAR and optical sensors. It also proved to be suitable for monitoring urbanization development. The encouraging results thus confirm the potential of combining information from SAR and optical sensors for timely urban area analysis, as required in cases of disaster management and planning in urban sprawl areas.

## 1. Introduction

Environmental monitoring of urban areas represents one of the main requests for citizens around the world and a topic challenge which has engaged the Earth Observation community in recent years. Continuous monitoring of urban areas is required in order to keep track of the loss of natural areas due to urban development and to support urban areas planning activities. Urban areas are particularly vulnerable, not only because of the concentration of population but also due to the interplay that exists between people, infrastructures and natural or man-made risks. Increasing numbers of disasters in densely-populated cities have demonstrated to the scientific community and to the local concerned parties and authorities the importance of better awareness for the protection of the environment and for the safety of the citizens. In applications related to urban monitoring, disaster and, generally speaking, civil protection management, reliable urban data and robust analytical technologies are becoming more and more crucial, mainly in developing countries. It is now widely agreed that Earth observation (EO) using remotely sensed imagery is a valuable source of information for urban sprawl and disaster monitoring. These issues and concerns are targeted by the LIMES project (Land and Sea Integrated Monitoring for European Security), established within GMES programme (Global Monitoring for Environment and Security. LIMES aims at defining and developing prototype information services to support management at EU and global level. One of the main objectives of the project is to improve the methodology for provision of rapid urban mapping products, especially in the fields of damage assessment and humanitarian relief and reconstruction. In the LIMES framework, we investigated the joint use of space borne optical and SAR sensors to characterize urban landscapes with the purpose of establishing an operational methodology for rapid urban mapping.

Although challenged by spatial and spectral heterogeneity of urban environments, optical and synthetic aperture radar (SAR) imagery seem to be suitable sources of reliable information about the multiple facets of urban environments [1, 2]. Some authors have attempted to explore the joint-use of SAR and optical sensors for urban characterization and monitoring [3]. In an overview of the literature covering urban remote sensing and data fusion issues, Gamba *et al.* [4], cited and discussed the main studies that exploited the synergism of multiple sensor data in general and SAR and optical sensors in particular.

The advent, over the last few years, of a third generation of very high spatial resolution (<5 m) SAR (i.e. Radarsat-2, CosmoSkymed, TerraSAR-X) and optical satellite sensors (i.e. Quickbird, Ikonos, SPOT-5) stimulated the development of urban remote sensing still further. The data produced by these satellites facilitate improved detection of subtle urban changes [5] and rapid expanding agglomerations and ‘edge cities’ of many developing countries [6]. It can also allow easier discrimination of the typology of urban landscapes especially in dense and heterogeneous cities. In their analysis of technical literature on the application of remote sensing to the study of human settlements, Donnay *et al.* [7] showed that the increasing availability of remote sensing sources of image data represents a serious challenge. While these offer new possibilities, each also presages new problems: despite the large number of urban remote sensing investigations that exploit multiple sensor data, doubts remain in some quarters about the potential for operational application of remote sensing to map and monitor urban areas. There is, for example, a concern about their robustness and reliability. At the same time, many of the remote sensing specialists are expending most of their efforts addressing the opportunities and problems posed by each new technological advance, rather than seeking operational solutions to the use of existing systems.

Finding simple yet effective operational approaches for urban extent extraction in optical and SAR images constitutes one of the main needs of urban practitioners and local governments. In the last decade, there have been a number of important methodological developments that attempted to fulfil these requirements. Some of the already available, computationally simple, semi-automatic procedures rely on morphological transformations [8], others on wavelet transform [9] or on textural analysis [10-12] of SAR and/or optical data. In the line of these approaches, we proposed and implemented an original approach for the analysis and classification of SAR and optical data. It is entirely automatic and properly designed for fast information extraction for the purpose of rapid urban mapping. Based on straightforward theoretical considerations, the methodology consists in a complete unsupervised procedure that can be applied on a pair of co-registered SAR/optical images for operational urban areas extraction. In Section 2 a detailed description of the basic idea underlying our algorithm is given. In Section 3, some experimental results are reported and analyzed. First, the methods' performance for urban areas delineation is assessed. Then, its capacity for monitoring urban expansion is evaluated. Section 4 presents some general comments and directions for future work.

## 2. Developed procedure

There is considerable confusion about the definitions used to describe urban areas. Therefore, prior to the presentation of the methodology, we propose to define the concept of urban areas in a pragmatic way in accordance with the information that could be derived from remote sensing data. In this study, an urban area refers to the geographical area of continuous built-up development. This geographical area exists in opposition to non–urban areas. This definition does not take in to account the criteria of population size nor the economical and functional levels.

To delimit the so-defined urban areas from SAR and optical sensors a three-step procedure is developed. It is organized as follows:

- texture analysis,
- fuzzy K-means clustering,
- information fusion.

**Figure 1.**
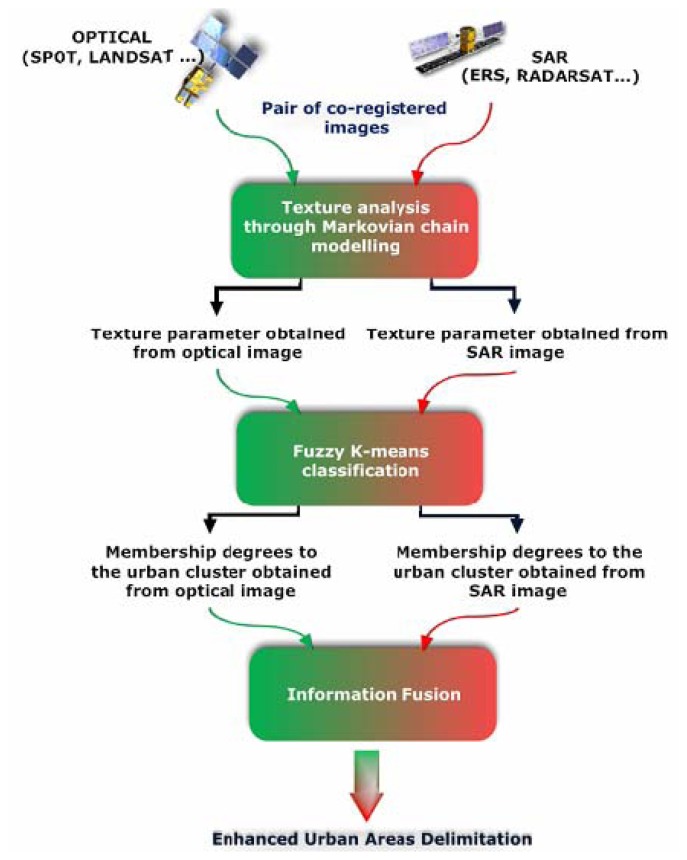
Flow chart of the three-step procedure for rapid urban mapping based on the synergy between SAR and optical sensors.

The conceptual workflow of the methodology is graphically shown in Figure 1. In a first step, the co-registered SAR and optical images are processed separately in order to analyze the texture through the estimation of the Gaussian Marko Random Field (GMRF) parameters. In a second step, a separate fuzzy K-means classification is applied on the resulting texture parameter images, in order to compute membership degrees for the urban cluster. In a third step, an information fusion scheme allows to aggregate the results provided by each sensor. The final decision is taken by selecting the class with the largest resulting membership value. A more detailed description of all the steps of the procedure is provided in the paragraphs that follow.

### 2.1. Texture analysis

Texture, which can be defined as a function of local variation of pixel intensities [13], is a useful image characteristic that has been successfully utilized in many automated image analysis algorithms. Many old and recent works on classification of optical and SAR satellite images in urban environments showed that textural features can yield high classification accuracies [10, 14, 15]. This is because urban environments are characterized more through their structure than through their spectral reflection properties or backscattering intensity.

Motivated by the success of texture-based image analysis algorithms, we use a similar strategy for our automated urban areas extraction system. Among the numerous model-based approaches described in the literature [16-18], we chose to analyze the texture through a four-connected GMRF model developed by Descombes *et al.* [19] for the following reasons:

- GMRF is quite a simple model requiring relatively few parameters and a reduced processing time which makes it suitable for rapid mapping purposes,
- with respect to the complexity of optical and SAR image textures, its parameters can discriminate these different textures, mainly those of urban areas,
- moreover the robustness of the employed technique for parameters estimation leads to an accurate delineation of the urban areas,
- finally the parameters are local mean independent.

We briefly recall the definition of the texture parameter. Full details can be found in [19] and [20]. This parameter is obtained from a multi-directional analysis of the texture. We consider eight directions in the discrete space and a Gaussian Markov Random Field for each direction. A pixel has two neighbours corresponding to the direction *d* for each model. The local conditional probability is then written as follows :

(1) $$P(X_{S}|X_{r},r\in V^{d}(s))=\frac{1}{Z_{V^{d}(S)}}\exp(-\beta^{d}(2+\lambda^{d})\times {\left(X_{s}-\frac{2m_{s}^{d}+\lambda^{d}\mu }{2+\lambda^{d}}\right)}^{2})$$

(2) $$=P(X_{S}|m_{S}^{d})$$

The local conditional distribution $P(X_{S}|m_{S}^{d})$ is a normal law defined as follows:

(3) $$P(X_{S}|m_{S}^{d})\equiv N(\frac{2m_{S}^{d}+\mu \lambda^{d}}{2+\lambda^{d}},\frac{1}{2\beta^{d}(2+\lambda^{d})})$$

where:

- *d* represents the direction,
- *X_s_* is the grey level value of pixel S, $V_{S}^{d}$ its neighbourhood in direction *d* and *X_r_* ∈ *V_s_*,
- *Z_Vd(s)_* is the partition function,
- μ is the local mean,
- β and λ are the texture parameters of the model.

It is shown in [20], that the conditional probability depends only on the mean of the 1D neighbourhood $m_{S}^{d}$. The texture descriptor we consider is given by the conditional variance:

(4) $${\sigma^{2}}_{p{\left(X_{S}|X_{r}\right)}^{d}}=\frac{1}{2\beta^{d}(2+\lambda^{d})}$$

It is estimated by the so called “comet tail” method in a window centred in pixel *S* [6]. The eight estimated values are then normalized with respect to the eight different directions in order to correct the bias introduced by anisotropy. Normalization is done by decimation that consists in computing marginal laws on sublattices [19, 20]. The eight normalized parameters are then combined into a single one (the texture parameter) which characterizes the urban areas: for each pixel, we classify the eight values in increasing order and we only keep the mean of the two median values (even number of parameters). Indeed these values are high for pixels inside urban areas, which are characterized by high variances in all directions. They are low for pixels in forests, fields, and water areas, which are characterized by low variances in all directions. To keep more than two median values (4 or 6) does not improve the detection of urban areas. The estimated texture parameter is robust and highly characteristic of urban areas. It is computed independently on the pair of co-registered SAR and optical images and used as a textural descriptor in the subsequent steps.

### 2.2. Fuzzy K-means clustering

The texture features images obtained at the previous stage are classified using a fuzzy K-means clustering method [21, 22]. The main advantage of such a classification is that it provides, for each pixel, membership degrees for the urban cluster thus offering the opportunity to handle the output for further processing, namely for the information fusion process. The Fuzzy K-Means (FKM) algorithm aims to find fuzzy partitioning of a given set, by minimizing the basic K-means objective function (*J*):

(5) $$J(Z;U,V)=\overset{c}{\underset{i=1}{\text{∑}}}\overset{N}{\underset{k=1}{\text{∑}}}{\left(\mu_{ik}\right)}^{m}{\left\|z_{k}-v_{i}\right\|}_{A}^{2}$$

where:

- ***Z*** is the data matrix,
- ***U*** =[*μ_ik_*] ∈ *M_fc_* is a fuzzy partition of ***Z***;
- ***V***= [v_1_, v_2_,…, v_c_], v_t_ ε **R***^n^* is a vector of cluster prototypes to be determined ;
- ‖*z_k_*– *v_i_*‖^2^ is a dissimilarity measure (Euclidean distance) between the sample *z_k_* and the center v*_i_* of the specific cluster *i* ;
- *A* is the distance norm matrix ;
- *m* ∈ (1, ∞) is a parameter that determines the fuzziness of the resulting clusters.
- The minimization of the objective function *J*(*Z*;*U*,*V*) under the constraint $\overset{c}{\underset{i=1}{\text{∑}}}\mu_{ik}=1$ leads to the iteration of the following steps :
  (6) $$\begin{array}{l} v_{i}^{\left(l\right)}=\frac{\overset{N}{\underset{k=1}{\text{∑}}}{\left(\mu_{ik}\right)}^{m_{Z_{K}}}}{\overset{N}{\underset{k=1}{\text{∑}}}{\left(\mu_{ik}\right)}^{m}},1\leq i\leq c\\ \;\text{and}\\ {\mu_{ik}}^{\left(l\right)}=\frac{1}{\overset{c}{\underset{j=1}{\text{∑}}}{\left(D_{ikA}/D_{jkA}\right)}^{\overset{2}{\;}/\underset{\left(m-1\right)}{\;}}}\text{otherwise}{\mu_{ik}}^{\left(l\right)}=0\text{if}D_{ikA}>0\text{and}\mu_{ik}\in \langle 0,1\rangle \text{with}\overset{c}{\underset{i=1}{\text{∑}}}\mu_{ik}=1 \end{array}$$

where: $D_{ikA}^{2}={\left\|z_{k}-v_{i}\right\|}_{A}^{2}$

The iteration (*l*) stops when the difference between the fuzzy partition matrices ‖*U*^(*l*)^ – *U*^(*l*−1)^‖ in the two previous iterations is lower than the termination criteria ε.

Urban areas delineation, can be considered as a 2-classes (urban/non-urban) classification problem for which two different classification results are available, one provided from the SAR image (*i*) and the other one by the optical image (*j*). Hence, for a given pixel *x*, the outputs of the fuzzy classification of the texture features images obtained from sources *i* and *j*, are respectively the sets of membership digrees:

(7) $$\pi_{i}(x)=\{\mu_{i}^{1},\mu_{i}^{2}\}\;\text{and}\;\pi_{j}(x)=\{\mu_{i}^{1},\mu_{i}^{2}\}\;\text{where}\;\mu_{i}(x)\in [0,1]$$

As a conclusion for every pixel, two fuzzy sets are computed {*π_i_*(*x*),*π_j_*(*x*)}. They constitute the input for the last fusion process.

### 2.3. Information fusion

This step is concerned with the combination of the information on urban areas issued from SAR and optical sensors so to improve the “urban/non-urban” classification results. Information fusion aims at exploiting redundancy, in order to increase global information and complementarity, to improve certainty and precision [23]. Information fusion is particularly flexible in the framework of fuzzy sets, due to existence of a variety of fuzzy combination operators, which may deal with heterogeneous information. Combination operators can be classified into three categories depending on their behaviour [24]. Let *x* and *y* denote two real variables representing the membership values to be combined and let *F* be the function acting on *x* and *y*, defining a combination or fusion operator:

(8) −FisconjunctiveifF(x,y)≤min(x,y)

This corresponds to a severe behaviour. The conjunctive operator is usually applied when searching for a redundancy between sources, or a common zone.

(9) −Fis disjunctiveifF(x,y)≥max(x,y)

This corresponds to an indulgent behaviour. The disjunctive operator is usually applied when searching for complementarity.

(10) −Fbehaves like acompromiseifx≤F(x,y)≤y

This corresponds to a cautious behaviour. Different types of fuzzy operators behave this way like the *Mean Operators* (i.e. the arithmetical mean (x+y)/2, the *Ordered weighted averaging operators* [25], etc.

Bloch [26, 27] proposed a new classification to describe these operators not only as conjunctive or disjunctive ones but also in terms of their behaviour with respect to the particular values of the information to be combined:

- *Context Independent Constant Behaviour Operators* (*CICB*): this class is composed of the operators which have the same behaviour whatever the values of information. They are computed without any contextual or external information. They are exclusive.
- *Context Independent Variable Behaviour Operators* (*CIVB*): they are context independent but their behaviour depends on the values of *x* and *y*.
- *Context Dependent Operators* (*CD*): they depend not only on *x* and *y* but also on global knowledge or measures on the sources to be fused (like conflict between sources or reliability of sources). For instance, it is possible to build operators which behave in a conjunctive way if the sources are consonant, in a disjunctive way if they are dissonant, and like a compromise if they are partly conflicting [28].

The latter are interesting for the combination of the results provided by SAR and optical sensors, since their adaptive features makes them able to combine information related to one class in one way (i.e. urban) and information related to another class in another way (i.e. non-urban). Besides they allow to obtain a fusion that is neither purely conjunctive, nor purely disjunctive avoiding this way a severe behaviour that may induce either a poor detection (in the case of the min operator) or a dramatic false alarm (in the case of the max operator).

For this study, the combination process is seen in the light of conflict between information provided by each sensor instead of reliability of sources. The selected fusion scheme has the advantage of being computationally simple and most importantly, it does not require any prior knowledge regarding the reliability of each sensor. In fact, the conflict indicates the degree of contradiction between sources.

The conflict according to [29] may be defined as 1-h with:

(11) $$h(\pi_{1}(s),\pi_{2}(s))=\underset{s}{\sup}\min[\pi_{1}(s),\pi_{2}(s)]$$

According to the level of conflict, the adaptive operator behaves as follows [24, 26]:

- it is conjunctive if the two sources have low conflict,
- it is disjunctive if the sources have high conflict,
- it behaves in a compromise way in case of partial conflict. The arithmetical mean belonging to the class of *Mean Operators* is used in this study.

The final binary decision (urban/non-urban) is issued at the end of the processing chain. It is based on the maximum of membership values after the combination step. In the proposed methodology, the final decision is taken by selecting the class with the largest resulting membership values.

## 3. Experimental results

### 3.1. Test sites and data set description

In this section, we present the application of the proposed procedure for the extraction of urban areas on two test sites of different terrain relief, urban settlement structure and land cover complexity. The first site is located in Bucharest (Romania) and covers an area of approximately 32 x 20 km. It is characterized by its flatness and the presence of a mixture of urban settlings and large agricultural fields. The second study area of 12 x 12 km is located in Cayenne (French Guiana) and is characterized by a heterogeneous relief and a mixed urban landscape with spontaneous settlements. Due to its location in tropical latitudes, Cayenne is known to be an area notorious for persistent cloud cover that presents a challenge to optical remote sensing operations.

To test the performance of the proposed procedure for urban areas extraction, we used two pairs of Radarsat-1 and SPOT-4 multispectral images acquired in 2001 (one pair for each site). We also tried to check the capacity of the methodology in monitoring urban development. The dataset, in this case, included a pair of ENVISAT–ASAR (APP product = Alternating Polarization Mode Precision Image with VV & VH polarizations) and SPOT-5 images acquired in 2006 over Cayenne. These images were used in combination with Radarsat-1 and SPOT-4 data from 2001 for analyzing the evolution of the urban areas between 2001 and 2006. Because the GMRF model requires single band data, texture feature extraction on SPOT data is performed using band B2, which is equivalent to the panchromatic mode of SPOT. All remotely sensed data both optical and SAR data required systematic corrections. The images were orthorectified. Speckle reduction, using a 5 x 5 Frost filter kernel, was applied to SAR data to reduce the data noise while retaining the information. Subsequently, 16 bit SAR data were converted to 8 bit data in order to compare with 8 bit optical data. Then, SAR data were resampled, resized and coregistered with the corresponding reference optical data. The main characteristics of the remote sensing data used in these experiments are presented in table 1.

### 3.2. Parameters involved

The main parameters of the three-step algorithm are set as follows:

For the texture analysis step, an estimation window of 15 × 15 pixels is used for the computation of the conditional variance in the eight directions. There is actually a trade-off in choosing the size of the window. This is due to the fact that as the window size increases, the texture feature is better estimated in terms of robustness in the statistics, but, the uncertainty zone between two different textures also gets larger, and the edges are not localized as accurately. It seems that a window size of 15 ×15 pixels is a good compromise.

For the fuzzy K-means clustering step, we set:

- *c* = the number of clusters = 2,
- *m* = the fuzzy exponent that determines the degree of fuzziness of the final solution; with the smallest value of 1, the solution is a hard partition, i.e., the results are not fuzzy at all. Most applications of fuzzy K-means use a value of m between 1 and 2; in our study, *m* = *2*,
- *ε* = the stopping criteria = 0.001(gives reasonable convergence),
- *l* = the maximum number of iterations = 5.

For the information fusion step, conflict values less than 0.5 are considered as non significant, over 0.8 are considered very high. In between these two values, we are in a situation of partial conflict.

For each of the three steps of the algorithm, the CPU time is calculated and presented in table 2.

### 3.2. Results analysis for urban areas extraction

The SAR/optical coupling methodology for automatic delineation of urban areas is tested against a single-source approach that lies on the individual use of SAR and optical data. We denote SS (Single Source), the approach in which a classical K-means algorithm is used for classifying SAR and optical driven texture images, without taking into account the final results integration step.

**Figure 2.**
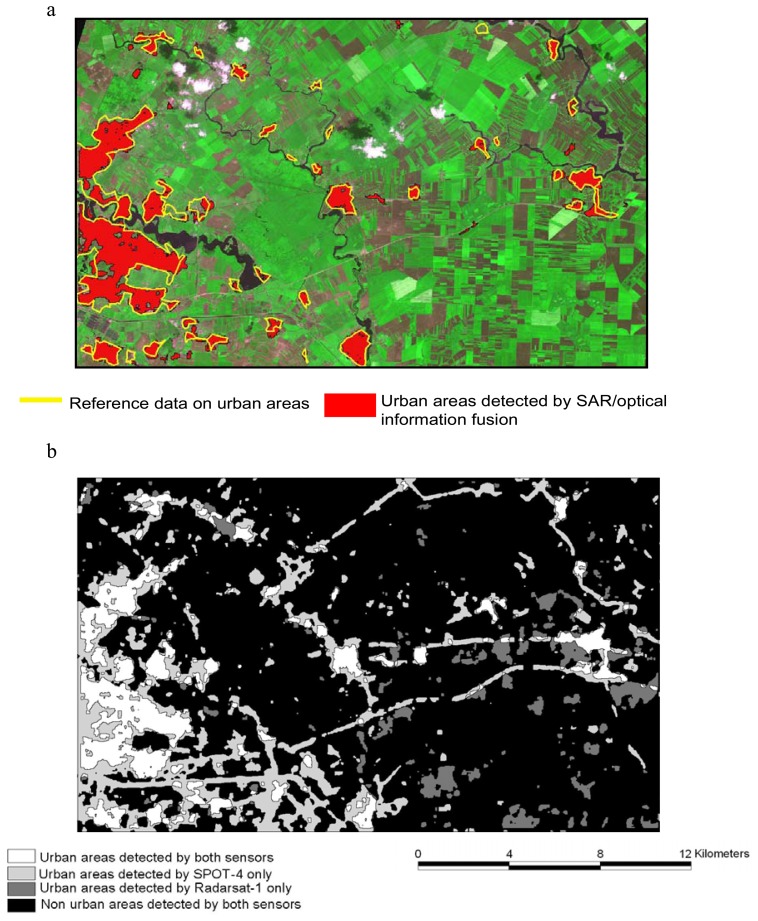
Results of the experimental tests over the Bucharest study area: a) the yellow contours correspond to the reference data obtained from visual interpretation of SPOT-4 imagery, the red polygons represent built-up areas resulting from the joint use of SPOT-4 and Radarsat-1 images according to the proposed approach; b) the greyscale map shows the results of the SS procedure applied separately on SPOT-4 and Radarsat-1 data. It evinces the complementary properties between the two images suggesting their use in a combined approach.

Unfortunately, suitable reference data for the first Bucharest site, showing the urban areas with a reasonable accuracy, are unavailable. Hence for this site, the validation of the results is conducted by visual interpretation and manual delineation of urban areas on the SPOT-4 image (yellow contours on Figure 2a). Conversely, a reference map, obtained by visual interpretation of an aerial photograph and by ground survey, is available for the Cayenne test site [30, 31]. This allows to quantitatively assess how much the proposed procedure is effective in improving the results obtained from the separate use of SAR and optical sensors.

Figure 2a shows the final delineation of urban areas (red polygons) obtained from the joint-use of Radarsat-1 and SPOT-4 images on Bucharest site compared to the reference data resulting from a computer-assisted visual interpretation of SPOT-4 imagery. For a better visual inspection of the result, delimited urban areas are overlaid on the multispectral SPOT-4 image. A qualitative comparison can be done between Figure 2a and Figure 2b where the results of the SS procedure, applied individually to SPOT-4 and Radarsat-1, are represented in the form of a greyscale map.

On the optical image, urban areas are characterized by a high variance. Nevertheless, such criteria lead to false detection, as it can be seen from the result in Figure 2b. Indeed, regions composed of small non-uniformly oriented objects such as row-cropped fields have identical responses as urban areas in some privileged directions. Besides, edges of roads, clouds and barren lands can also have high values for the texture parameter leading to some false detections. On the other hand, the false alarms on SAR image correspond to areas where backscattering varies a lot, like fields with rough bare soils or with high soil moisture content. In Figure 2b we may also notice the complementarity between the two types of images and the importance of taking advantage from the strengths of SAR and optical sensors. The combination of the information derived from the pair of SAR and optical images allows to reduce the uncertainty in urban areas extraction by exploiting the complementarities between the two sources as evidenced in Figure 2a. The remarkable improvement is noticeable in the precise limits of urban areas and the reduction of false alarms.

In Table 3a a quantitative evaluation of the results obtained from the joint use of SAR and optical sources compared to the SS approach is presented. The results are expressed in terms of the total surface covered by urban areas in km^2^ in comparison to the reference area obtained by visual interpretation of the SPOT-4 image. The table highlights the significant improvement in urban areas estimation resulting from the use of the SAR/optical information fusion approach. The result obtained from the sole use of Radarsat-1 imagery shows an underestimation of the real urban areas extent of almost 16.1 km^2^. The underestimation is essentially related to the presence of some low density urban areas that do not face the radar beam and accordingly have a low return signal. On the other hand, an overestimation of urban extents of approximately 13 km^2^ is achieved by the application of the SS approach to SPOT-4 image. The main reason for that is the existence of several row cropped fields with highly varying reflective properties resulting in high responses of the texture parameter like in urban areas. The joint-use of Radarsat-1 and SPOT-4 images following the proposed methodology, allows to considerably reduce the areal differences between the available reference and the automatically mapped urban areas.

**Figure 3.**
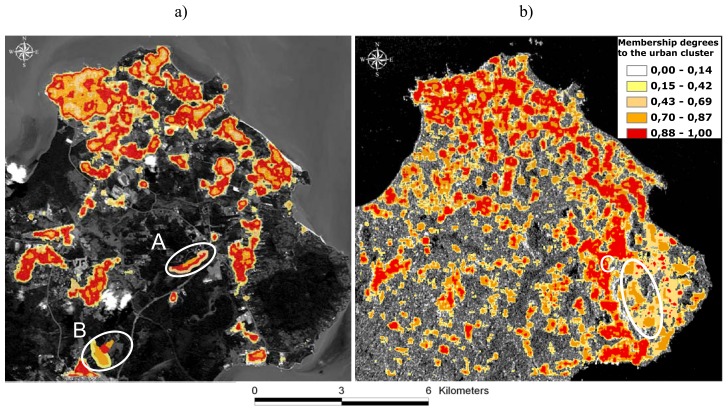
Results of fuzzy K-means classifications of urban textures extracted from: a) SPOT-4 and b) Radarsat-1 on Cayenne city. Each colour class corresponds to a range of degrees of membership to the urban cluster.

For the Cayenne site, the results of fuzzy K-means classifications of urban textures extracted from SPOT-4 and Radarsat-1 images are represented on Figures 3a and 3b, respectively. The different colours represent the fuzzy membership values to the urban cluster in the range (0-1): the higher the membership degree, the more likely the pixel could be treated as belonging to the urban cluster. On optical data, certain urban built-up materials can be easily confused with non asphalt roads, bare soils, sparsely vegetated areas, muddy coastal sediments and clouds. The ellipses overlaid on figure 3a highlight some of these confusions. Ellipse A is an example of incorrect assignment of high membership values to sparsely vegetated areas. Likewise, ellipse B is an illustration of abusive false assignment of bare soils to the urban cluster. On SAR data, the above cited confusions are overcome. However, new types of false assignments may be encountered. Ellipse C represents an example of the confusions associated with the SAR scene. It corresponds to regrowth forests incorrectly identified as urban areas with relatively high membership values (0.7 to 0.87). In this south-eastern part of Cayenne, regrowth forests are located on steeply sloping areas facing the radar beam. They appear bright in SAR imagery and can be easily mistaken with urban settlements. These results highlight the limitations of using SAR and optical data sources individually and the importance of exploiting the information provided by each sensor following the information fusion scheme.

Table 4 shows a quantitative evaluation of the results obtained from the joint use of SAR and optical sources compared to the SS approach. The “reference area” of urban areas in the window under study is estimated to be around 24.3 km^2^. A first comment to this table is that the sole use of SAR data in a SS approach leads to an underestimation of the real extent of urban areas of 6.5 km^2^. This is essentially related to the highly cluttered nature of the urban areas in Cayenne city where changes in street orientation and in building density induce strong variations in the backscattering echo and hence in the texture parameter. For instance, significant missed detections were observed in high density urban areas in the south-western part of Cayenne. This may be explained by the fact that when the urban density is large, some buildings may be hidden from the radar illumination by other buildings. This shading results in a weak backscatter. Besides, high-density squatter settlements are characterized by flat roofs made of metal plates. Hence, the roof faces appear dark on SAR imagery, because the signal is mainly reflected away from the sensors. The strong spatial heterogeneity and composition of the urban environment in Cayenne city adds evidence on the complexity of detecting urban settlements and explains the significant underestimation of their real extent.

**Figure 4.**
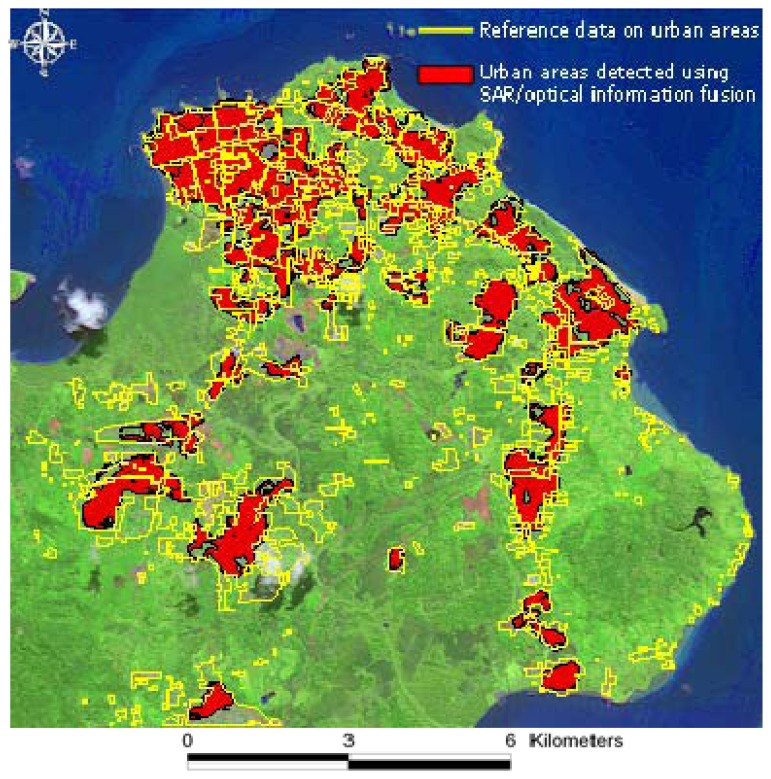
Final result of automatic urban areas delimitation using the proposed SAR/optical information fusion. Reference data on urban areas is also shown for visual validation of the results. SPOT-4 natural color is used as background image (B4: Red; B3: Green; B2: Blue).

On the contrary, the SS approach applied to optical data results in an overestimation of urban areas of almost 6 km^2^. The advantage of the joint use of SAR and optical data is evident in the reduction of the difference between the “reference area” and the one resulting from the automatic rapid urban mapping approach. The proposed procedure not only allows reducing the deviation from reference to 4 km^2^, but also provides well-located limits of urban areas and few false alarms as depicted in Figure 4. However, in terms of overall performance, our approach undervalues the extent of urban areas. Underestimation is mainly observed in peri-urban areas located in the western and south-western sectors of Cayenne.

### 3.3 Results for monitoring the spatial extension of urban growth

To illustrate the application of the extraction methodology to urban growth monitoring, the pair of ENVISAT ASAR and SPOT-5 images acquired on 2006 over Cayenne were used in combination with the above obtained results on SAR and optical data from 2001. In the case of ENVISAT ASAR data, only the VV polarization was processed with the SAR/optical information fusion approach, because it provided better results than the cross-polarization for the extraction of urban areas. The total built-up area obtained in 2006 by the SAR/optical information fusion approach applied on the ENVISAT ASAR and SPOT-5 data is around 23.5 Km^2^. It reflects an increase in urbanization when compared to the result obtained in 2001 (20.3 Km^2^).

**Figure 5.**
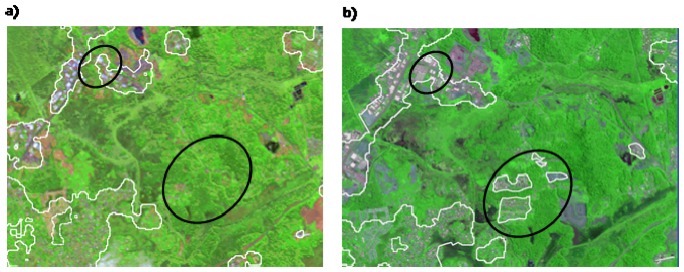
Extraction of urban areas from 2001 (a) and 2006 (b) in Cayenne. The zoom in Figure 5a represents the result obtained with the SAR/optical information fusion applied on Radarsat-1 and SPOT-4 imagery, whereas the zoom of Figure 5b corresponds to the result obtained thanks to the combination of ENVISAT ASAR (VV polarization) and SPOT-5 imagery. The ellipses refer to the newly built-up areas that were successfully identified in the 2006 images.

In Figure 5, we notice that the extraction methodology succeeded in delimiting the newly built-up areas. The two zoom windows, one for 2001 and one for 2006, clearly show the efficiency of the extraction methodology in delineating new urban settlements and its suitability for monitoring urbanization development. A comparison with the reference map shows that all the built-up areas reported in the ground-truth map are recognized as such thus confirming the benefit of using SAR images in combination with optical data for accurate delineation results (Table 5).

## 4. Conclusions

In this paper, we have proposed an automatic method for operational rapid urban mapping. The developed approach takes advantage of the complementary properties between SAR and optical sensors for an enhanced delimitation of urban areas. The experimental part of this paper was performed on two pairs of Radarsat-1 and SPOT-4 images acquired over two different test sites. The performance of the proposed procedure was also tested against a separate use of SAR and optical sensors. First, we evaluated the method in a flat area, close to Bucharest (Romania), including mainly dense urban settlements surrounded by large agriculture fields. When dealing with this type of urban environment, optical imagery offers limited information on the extension of urban areas. This is mainly due to similar high variances between urban areas and some non-uniformly oriented objects like row-cropped fields that have an anisotropic behaviour. In SAR imagery, on the other hand, the main false detections are due to high variances obtained over rough or wet bare soils that return a strong signal and appear bright in a radar image. Second, we tested the method on Cayenne city, an area with great local elevations and several significant steeply sloping sectors covered by spontaneous urban settlements. The analysis revealed that the sole use of optical imagery may result in false detections, mainly associated with the presence of non asphalt roads, bare soils, sparsely vegetated areas and clouds. Alternatively, on SAR imagery urban areas may be easily recognized because of their strong and rarely anisotropic backscatter. However, SAR backscatter from an urban environment is highly dependent on the radar frequency, polarisation and viewing geometry. Therefore SAR imagery allows detecting urban features in a complementary way, but it can also become blind towards other buildings and structures depending on the viewing geometry, the incidence angle and the urban fabric.

These findings demonstrate that the limitations of SAR and optical sensors for urban areas extraction may be compensated by appropriately combining the information extracted from each sensor. The results obtained from the joint-use of SAR and optical data have consistently demonstrated the validity of our proposal. In the two experiments, false detections were reduced and the final results showed accurate delineation of urban areas. Nevertheless, even in the absence of simultaneous SAR and optical images, the urban extraction methodology could be applied using the SS approach (Signle Sensors approach) while still providing satisfactory results.

We have presented a framework for urban areas extraction that proved to be suitable for monitoring urban extension. This is of great value for planning in urban sprawl areas where up-to-date information is lacking because of the rapid pace of house construction and residential development.

In terms of computational cost, the methodology is time-efficient. The IDL source codes have not been yet optimized. Currently our unoptimized implementation runs on a 2 GHz Pentium IV-class machine. The speed for an average 3,000 x 3,000 pixels is around 20 minutes, thereby proving the suitability of our approach for rapid mapping purposes.

Presently, we are extending the use of this approach for urban areas extraction on very high spatial optical images combined with SAR images in different configurations (different spatial resolutions, incidence angles, polarizations). The purpose is to determine the optimal configurations that allow to fully exploit the advantages of SAR and optical data for enhanced urban areas extraction. Future exploratory works will include investigations on alternative fuzzy combination rules for the information fusion step. For instance, it would be interesting to examine the effect of using other Contextual Dependent Operators [26] such as the incorporation of knowledge regarding reliability of one given source or integration of some spatial information. The idea would be to test different fusion strategies and to compare their respective contributions in improving the detection performances. Other possible extensions to this work could be the integration of multispectral information and other textural features (i.e. textural parameters extracted from the Grey Level Co-occurrence Matrix or from Gabor Filters). That way we can integrate much complementary information from SAR and optical sensors for the optimized rapid mapping process.

## Figures and Tables

**Table 1.** Characteristics of the image data set used in the experiments. * refers to the dataset used for testing the methods' performance for urban extraction; † refers to the dataset used for testing the methods' performance for monitoring urban expansion.

	Bucharest (Romania) *		Cayenne (French Guiana) *		**Cayenne (French Guiana)** †	
**Source**	kalideos Database		Espace unit IRD Database		Espace unit IRD Database	
**Sensor Type**	Radarsat -1	SPOT-4 (B2)	Radarsat-1	SPOT-4 (B2)	ENVISAT ASAR	SPOT-5 (B2)
**Date of acquisition**	03/05/2001	03/05/2001	01/05/2001	02/07/2001	29/03/2006	30/08/2006
**Incidence angle (°)**	16.7		39		36.8	
**Pixel size (m)**	12.5 x 12.5	20 x 20	12.5 x 12.5	20 x 20	12.5 x 12.5	10 x 10
**Coregistration RMSE (pixels)**	0.27		1.12		1.31	

**Table 2.** CPU time for each step of the algorithm calculated for the two tests (2 GHz Pentium IV).

	**Image size**	**CPU Texture analysis**	**CPU FKM**	**CPU Information fusion**
**Bucharest (2001)**	1,600 * 1,000	3 min 20 s	4 min 12 s	2 min 10 s
**Cayenne (2001)**	600 * 600	1 min 02 s	2 min 11 s	1 min 45 s
**Cayenne (2006)**	600 * 600	4 min 15 s	5 min 15 s	3 min 51 s

**Table 3.** Comparison of urban mapping capabilities for the SS approach and the proposed SAR/optical information fusion approach, in terms of spatial extent of urban areas and deviation to reference area in the case of Bucharest site. The reference area, obtained from visual interpretation of the SPOT-4 image, is estimated to be around 46.5 km^2^.

**BUCHAREST SITE**	**SS approach**		**SAR/optical information fusion**	**Reference area**
**Extent of urban areas (km^2^)**	Radarsat -1	SPOT-4 (B2)	44.04	46.5
	30.4	74.3		
**Deviation from reference area (km^2^)**	- 16.1	+ 13.3	- 2.46	NA

**Table 4.** Comparison of urban mapping capabilities for the SS approach and the proposed SAR/optical information fusion approach, in terms of spatial extent of urban areas and deviation to reference area in the case of Cayenne site in 2001. The reference area, obtained from ground survey and visual interpretation of aerial photos, is estimated to be around 24.3 km^2^.

**CAYENNE SITE (2001)**	**SS approach**		**SAR/optical information fusion**	**Reference area**
**Extent of urban areas (km^2^)**	Radarsat -1	SPOT-4 (B2)	20.3	24.3
	17.8	30.2		
**Deviation to reference area (km^2^)**	- 6.5	+ 5.9	- 4	NA

**Table 5.** Comparison of urban mapping capabilities for the SS approach and the proposed SAR/optical information fusion approach, in terms of spatial extent of urban areas and deviation to reference area in the case of Cayenne site in 2006.

**CAYENNE SITE (2006)**	**SS approach**		**SAR/optical information fusion**	**Reference area**
**Extent of urban areas (km^2^)**	ENVISAT ASAR	SPOT-5 (B2)	23.5	24.3
	20.3	34.6		
**Deviation to reference area (km^2^)**	- 4	+ 10.3	- 0.8	NA
